# A brain mass in a patient with Behcet’s disease: a case report

**DOI:** 10.1186/s13256-015-0694-5

**Published:** 2015-09-30

**Authors:** Ahmad S. Alfedaghi, Y. Masters, M. Mourou, O. Eshak

**Affiliations:** Al-Adan Hospital, Hadiya, Kuwait

## Abstract

**Introduction:**

This case report describes an uncommon presentation of Behcet’s disease which manifested as neuro-Behcet’s disease. Although it is not the first reported case in the medical literature, it is a possible differential in a patient presenting with a brain tumor. Since the diagnosis of neuro-Behcet’s disease depends largely on the clinical picture and medical history, it should be considered prior to opting for invasive diagnostic methods.

**Case presentation:**

Our patient is a 36-year-old white man from Kuwait. He presented with acute onset of headache, vomiting, and right-sided weakness. Magnetic resonance imaging of his brain showed a mass in the brain stem. He then revealed that he had a history of recurrent painful oral and genital ulcers for the past 10 years, which suggested a diagnosis of Behcet’s disease. A brain biopsy was recommended by a neurosurgeon at the time, but the patient refused the procedure. After initiating steroid therapy, the mass began to regress and, eventually, was undetectable on subsequent imaging of his brain.

**Conclusions:**

This case of neuro-Behcet’s disease reflects the need to consider this diagnosis in a patient of less than 40 years of age presenting with a suspected brain tumor. This may delay the need for invasive diagnostic methods, especially if such methods are not desired by the patient. In the management of suspected neuro-Behcet’s disease, initiating steroid therapy and measuring the response is a reasonable option before seeking a definitive diagnosis via brain biopsy. If the response to steroids is minimal then a brain biopsy should be performed.

## Introduction

Behcet’s disease is an idiopathic chronic relapsing vasculitis characterized by recurrent oral aphthous ulcers, genital ulcers and uveitis. It is thought to be an autoimmune disease that can involve any organ system including the skin, lungs, joints, brain, cardiovascular system, and even the gastrointestinal and urinary tract. This disease usually manifests at an average age of 25 to 35 years, and the incidence of this disease is highest among people of Middle Eastern and Japanese descent [1]. Neurological manifestations tend to affect 10 to 30% of patients and usually occur late in the disease [2]. These include cerebral thrombosis in the arterial or venous systems, meningoencephalitis, demyelination, memory loss or behavioral changes and many others. It is uncommon for a patient with Behcet’s disease to present with an inflammatory pseudo-tumor in the brain [1]. This case report discusses an unusual case of neuro-Behcet’s disease describing its clinical features, diagnostic modalities used and management. A similar case has been discussed in the literature with similar findings on magnetic resonance imaging (MRI) of the brain, but with a different clinical presentation [2].

## Case presentation

A 36-year-old white Kuwaiti man presented with a 2-day history of headache and vomiting. The headache was localized to his left temporal area, continuous and pressing in nature. It was exacerbated by movement of his head, and associated with two episodes of vomiting. On physical examination, he was vitally stable; a neurological examination showed mild right-sided arm and leg weakness (4/5) with mild right-upper facial nerve palsy. Sensory and fundus examinations were normal, and examination of the other systems was unremarkable.

**Fig. 1:**
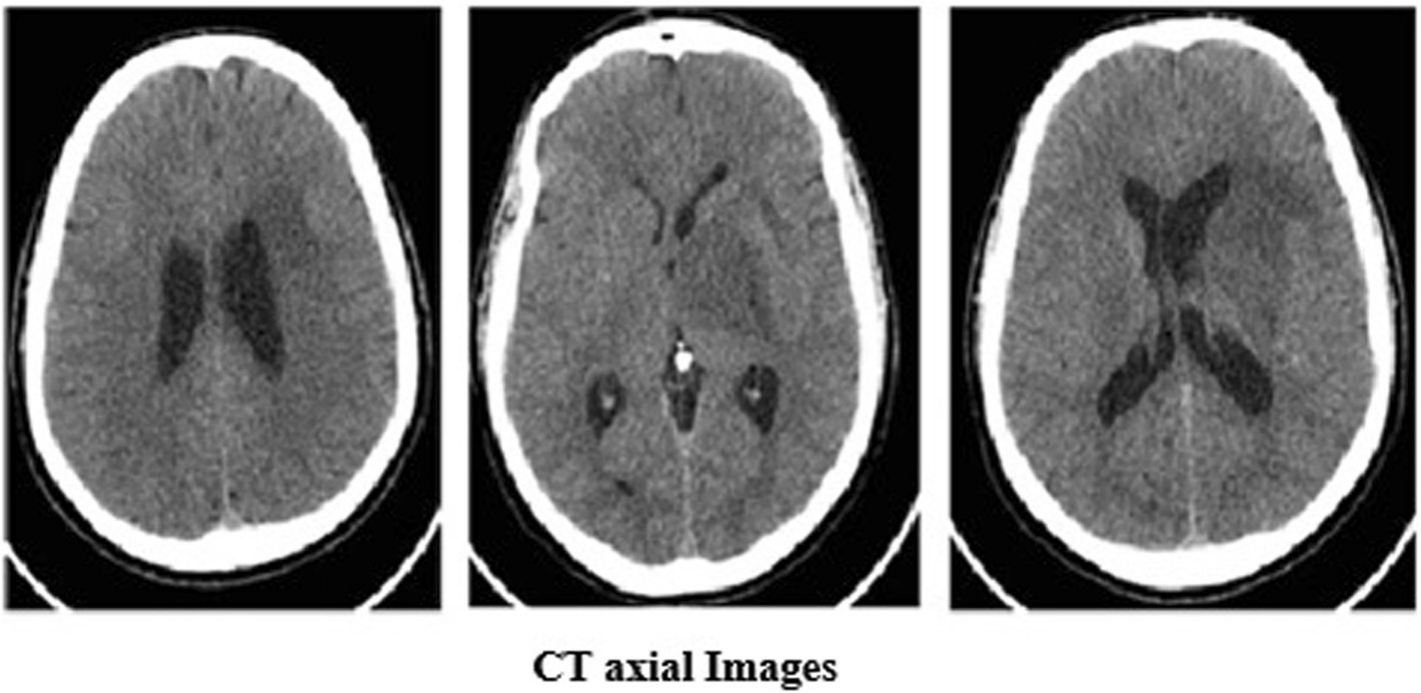
Brain computed tomography (axial images). Left temporoparietal hypodensity extending into left cerebral peduncle with faint central hyperdensity with mass effect and midline shift of 5mm

**Fig. 2:**
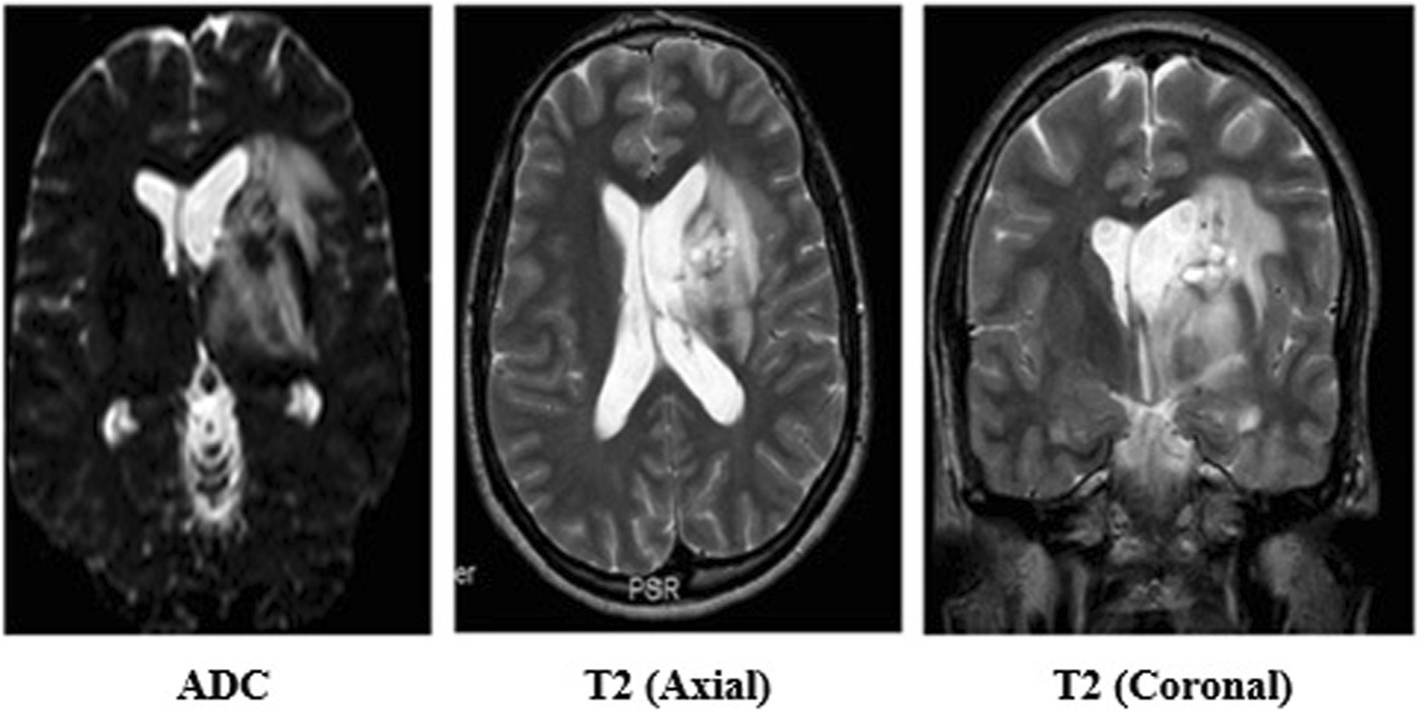
Brain magnetic resonance imaging. Apparent diffusion coefficient (ADC) and T2 images showed altered signal in the left basal ganglia extending to the left thalamus, midbrain and pons with the lesion causing mild fullness of the ipsilateral lateral ventricle due to compression of the left foramen of Monro

**Fig. 3:**
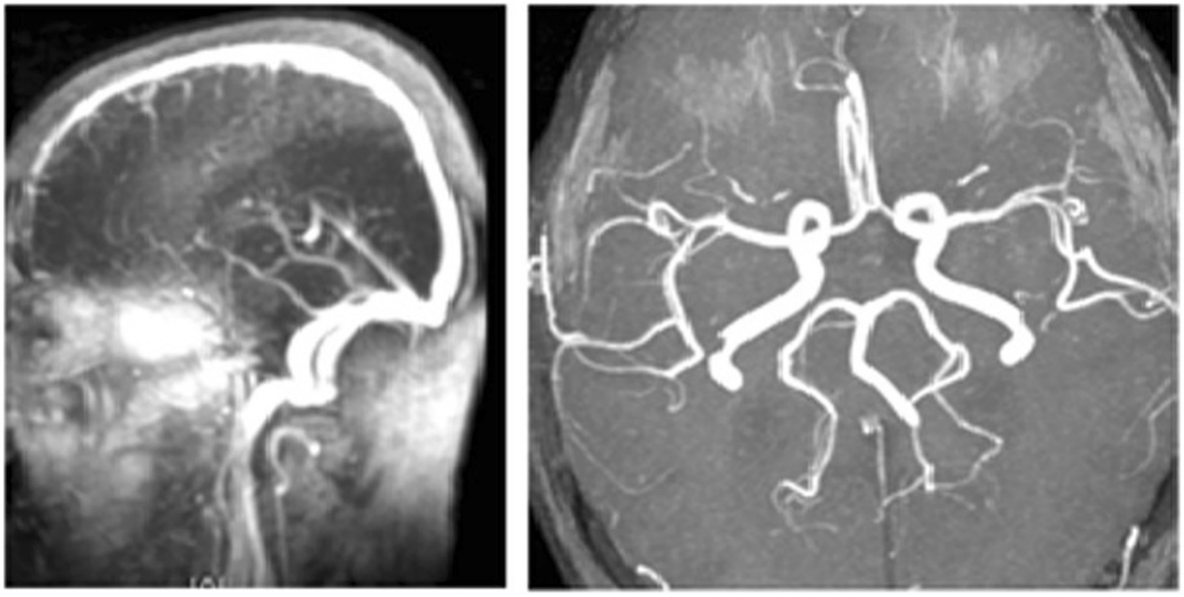
Normal magnetic resonance angiography and magnetic resonance venography

Laboratory testing which including a complete blood count, renal function test, liver function test, serum electrolyte, inflammatory markers such as erythrocyte sedimentation rate and C- reactive protein, viral serology, including human immunodeficiency virus (HIV), and blood cultures did not yield any abnormalities. An initial computed tomography (CT) of his brain showed an ill-defined left temporoparietal hypodensity with a faint central hyperdensity with mass effect and midline shift (Fig. 1). Due to the high suspicion of cerebral venous/arterial thrombosis, MRI of his brain as well as magnetic resonance venography (MRV) and magnetic resonance angiography (MRA) were performed. Imaging showed a mass involving his left thalamus, midbrain, and pons. The lesion resulted in mild fullness of the ipsilateral lateral ventricle due to compression of left foramen of Monro with midline shift (Fig. 2). MRV and MRA were unremarkable (Fig. 3). After reviewing his past medical history, he reported heavy cigarette smoking with recurrent episodes of painful mouth and genital ulcers which were treated with low-dose steroids for the last 10 years. His family history was unremarkable for chronic or inherited disease and there was no obvious precipitating factor for his current condition. After consulting the neurologist, the space-occupying lesion in his brain was suspected to be a brain tumor and a brain biopsy was advised; however, the patient refused and preferred medical treatment instead.

**Fig. 4:**
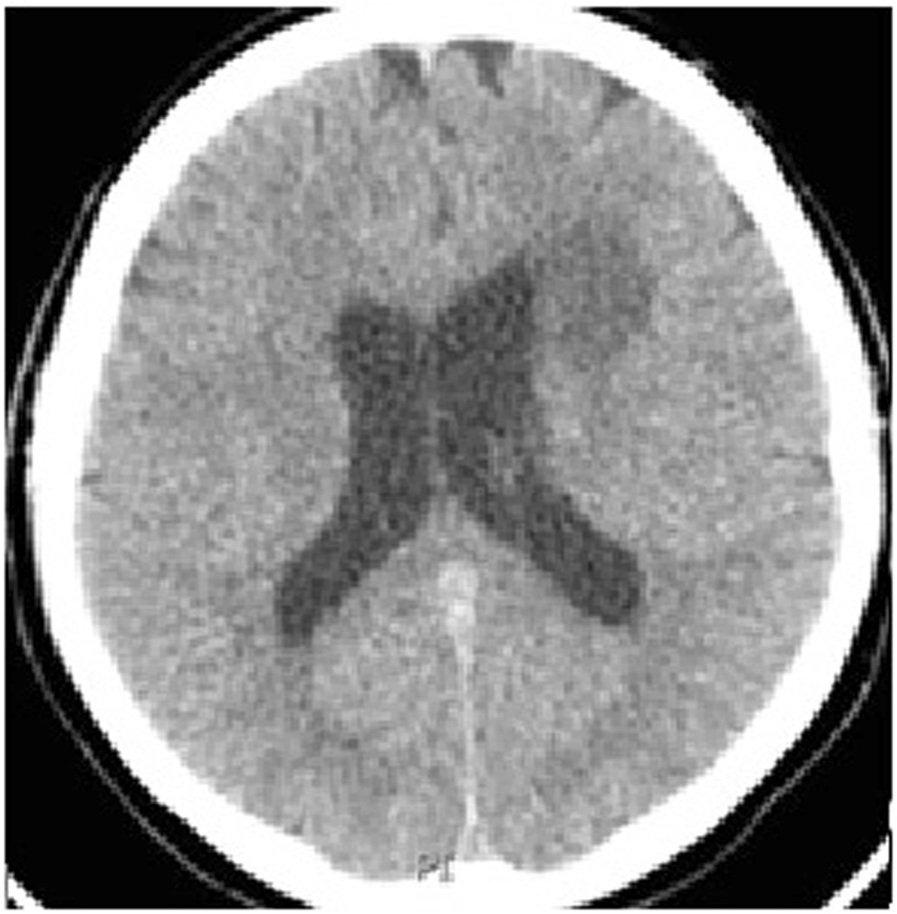
Follow-up computed tomography image (after 1 month) with more than 50% reduction in the size of the mass with minimal brain edema

The patient was treated with methylprednisolone, 1 gram once daily, for 7 days followed by high-dose prednisone, 60mg per day, and the lesion slowly regressed over the next month (Fig. 4). His symptoms improved dramatically and the lesion was undetectable on follow-up brain MRI after 3 months of therapy indicating that the lesion was most likely inflammatory in origin. Even though brain lymphoma can present in a similar manner and respond to steroid therapy, the classic history of recurrent oral and genital ulcers, the clinical context and the significant response to steroids, indicated that the mass is more likely to be related to neuro-Behcet’s disease.

## Discussion

### Clinical description

Neuro-Behcet’s disease is not uncommon but usually occurs late in the disease. It can affect both the central and peripheral nervous systems and the clinical manifestations of the disease depend on which part of the brain is affected. If the disease involves the central nervous system, it can result in lesions in the brain hemisphere manifesting as aphasia, limb weakness or numbness or it can affect the brainstem causing problems with vision, hearing or, sometimes, change in mental status and respiratory arrest. The spinal cord can be affected resulting in bilateral limb paresis with problems with urination and defecation. Neuro-Behcet’s disease can also affect the brain in other ways such as in dural sinus thrombosis resulting in intracranial hypertension, headache, papilledema, seizures and nerve palsy. Arterial occlusion or aneurismal dilatation are less common than venous involvement but have a worse prognosis and can present with signs and symptoms of cerebrovascular accident [3]. In our case, the patient presented with acute onset of headache, vomiting and mild weakness suggestive of increased intracranial pressure despite normal imaging of the venous and arterial system of his brain. Imaging revealed a mass in his brain stem, which was thought to be a brain tumor.

### Diagnostic modalities

According to Chae *et al*., MRI findings of neuro-Behcet’s disease typically present as round or linear hypointensities in T1-weighted images or as high signal intensities in T2-weighted images affecting the cerebral peduncles, pons, thalamus and basal ganglia [4]. Rarely, it can be viewed as an atypical space-occupying lesion mimicking lymphoma, primary or secondary brain tumor or even an abscess. Some authors do recommend stereotactic biopsy to rule out malignancy, but it is unnecessary for the diagnosis of neuro-Behcet’s disease. A biopsy would show perivascular infiltration of leukocytes and microglia, oligodendroglial degeneration, diapedetic bleeding, and areas of necrosis [2]. In our patient, imaging of his brain showed atypical features of neuro-Behcet’s disease and a biopsy was recommended by neurosurgery; however, the patient refused and decided on medical treatment before employing invasive measures. Even though brain lymphoma was a possibility, the classic history of recurrent oral and genital ulcers, the clinical context, acute symptoms and the significant response to steroids, made the possibility of lymphoma less likely especially as our patient is HIV negative and because of his blood culture results. There are multiple case reports that presented similarly to this case and they were proven by biopsy to be neuro-Behcet’s disease, so in the context of Behcet’s disease the clinical picture is more important that a brain biopsy [2].

### Management

The gold standard for treatment of neuro-Behcet’s disease is methylprednisolone administered as 1 gm per day for 1 week, followed by prednisone 0.5 to 1mg/kg/day with gradual tapering over the next 2 to 3 months. This treatment modality is considered the standard by many physicians. Other treatment modalities include azathioprine, methotrexate, cyclophosphamide, and anti-tumor necrosis factor (TNF) drugs as well as interferon-α, chlorambucil, and mycophenolate mofetil. These treatment modalities have not been studied well in the treatment of neuro-Behcet’s disease [2]. Even though neuro-Behcet’s disease is known to be associated with poor prognosis, the patient in the case described improved dramatically with pulse steroids and is currently on a small dose of prednisone with regular follow-up. We suggest that in a background of history indicative of Behcet’s disease, in a patient refusing a brain biopsy, delaying a biopsy and treating the patient with steroids with good follow-up as well as measuring the response to treatment is a plausible option. If the response is suboptimal then a biopsy is advised.

## Conclusions

Behcet’s disease is a form of vasculitis that may affect any organ system including the central nervous system. Neurological manifestations of this disease usually occur late in the disease course and are severe. It can affect the parenchyma, venous or arterial system of the brain resulting in a variety of symptoms and signs depending on the affected site. MRI is the modality of choice and typically shows lesions in the brain stem or, atypically, a space-occupying lesion in the brain. Our case presented with an acute onset of headache, vomiting and limb weakness with an atypical brain mass on imaging, which responded significantly to steroid therapy. This suggests the importance of thorough history taking in any patient with suspected brain tumor. In a background of history indicative of Behcet’s disease, in a patient refusing a brain biopsy, delaying a brain biopsy and treating the patient with steroids with good follow-up as well as measuring the response to treatment is a plausible option. If the response is suboptimal then a biopsy is advised.

## Consent

Written informed consent was obtained from the patient for publication of this case report and accompanying images. A copy of the written consent is available for review by the Editor-in-Chief of this journal.
